# ANGPTL8 deficiency attenuates lipopolysaccharide-induced liver injury by improving lipid metabolic dysregulation

**DOI:** 10.1016/j.jlr.2024.100595

**Published:** 2024-07-15

**Authors:** Ying Feng, Shan Luo, Chen Fang, Shinan Ma, Dandan Fan, Yanghui Chen, Zhuo Chen, Xiang Zheng, Yijun Tang, Xiaobei Duan, Xingling Liu, Xuzhi Ruan, Xingrong Guo

**Affiliations:** 1 Hubei Key Laboratory of Embryonic Stem Cell Research, Hubei Provincial Clinical Research Center for Umbilical Cord Blood Hematopoietic Stem Cells, School of Basic Medical Sciences, Taihe Hospital, Hubei University of Medicine, Shiyan, Hubei, China; 2 Department of Endocrine rheumatology, Taihe Hospital, Shiyan, Hubei, China; 3 Biomedical Research Institute, Hubei University of Medicine, Shiyan, China; 4 School of Biomedical Engineering, Hubei University of Medicine, Shiyan, Hubei, China; 5 Department of Neurology, Wuhan NO.1 Hospital, Wuhan, China; 6 Department of Critical Care Medicine, Taihe Hospital, Shiyan, Hubei, China

**Keywords:** liver injury, ANGPTL8, lipid deposition, PGC1α/PPARα pathway

## Abstract

Liver injury is closely related to poor outcomes in sepsis patients. Current studies indicate that sepsis is accompanied by metabolic disorders, especially those related to lipid metabolism. It is highly important to explore the mechanism of abnormal liver lipid metabolism during sepsis. As a key regulator of glucose and lipid metabolism, angiopoietin-like 8 (ANGPTL8) is involved in the regulation of multiple chronic metabolic diseases. In the present study, severe liver lipid deposition and lipid peroxidation were observed in the early stages of lipopolysaccharide (LPS) induced liver injury. LPS promotes the expression of ANGPTL8 both in vivo and in vitro. Knockout of *Angptl8* reduced hepatic lipid accumulation and lipid peroxidation, improved fatty acid oxidation and liver function, and increased the survival rate of septic mice by activating the PGC1α/PPARα pathway. We also found that the expression of ANGPTL8 induced by LPS depends on TNF-α, and that inhibiting the TNF-α pathway reduces LPS-induced hepatic lipid deposition and lipid peroxidation. However, knocking out *Angptl8* improved the survival rate of septic mice better than inhibiting the TNF-α pathway. Taken together, the results of our study suggest that ANGPTL8 functions as a novel cytokine in LPS-induced liver injury by suppressing the PGC1α/PPARα signaling pathway. Therefore, targeting ANGPTL8 to improve liver lipid metabolism represents an attractive strategy for the management of sepsis patients.

Sepsis is a life-threatening multiple organ dysfunction caused by an impaired host response to infection (1, 2) and is closely associated with high mortality in intensive care units (ICUs) (3, 4). Hospitalized patients are primarily infected by gram-negative bacteria, which can release the cell wall component lipopolysaccharide (LPS) to activate immune pathways (5). The innate immune system is recognized as the first line of defense against pathogen invasion, but overactivation of the innate immune system can lead to cytokine storms, multiple organ dysfunction, and even death (6). It has been demonstrated that the liver plays an important role in immune defense against pathogen invasion, and liver injury is one of the deadliest complications of sepsis (7, 8).

Liver injury may be one of the major causes of the initiation and progression of sepsis-associated multiple organ dysfunction syndrome (MODS) (9). In addition to its digestive function, the liver plays an irreplaceable role in the maintenance of metabolic and immune homeostasis (10, 11, 12). Although the incidence of liver injury is lower than that of lung injury, kidney injury and other organ injury during sepsis, liver injury is an important factor affecting the severity and prognosis of sepsis patients (13). However, the pathological mechanism of liver injury during sepsis is not yet fully understood, and methods for the early identification of liver dysfunction and effective treatment for liver injury in sepsis patients are lacking. Therefore, exploring pathogenesis is highly important for the early recognition and treatment of sepsis-related liver injury.

Changes in lipid metabolism and activation of lipid signaling pathways are components of the complex pathophysiological environment of sepsis (14, 15). An overactivated immune response, mitochondrial damage, and suboptimal feeding lead to a state of hunger (16, 17). First-line energy-supplying molecules such as glycogen and glucose are quickly depleted and supplemented by lipids released from adipose tissue, a process known as lipolysis (18). The free fatty acids (FFAs) produced by lipolysis are mainly taken up by the liver and undergo β-oxidation to provide energy and ketone bodies (19, 20). However, glucocorticoid receptors, peroxisome proliferator-activated receptors (PPARα), and other key factors of lipid metabolism are inactivated in the pathological state of sepsis, which leads to the accumulation of harmful metabolites in the liver, such as free fatty acids and lactic acid (21). Therefore, it is important to elucidate the mechanism of lipid metabolism perturbations in LPS-induced liver injury.

ANGPTL8 is a secreted protein that is highly expressed in liver and adipose tissue and is related to glucose and lipid metabolism and the inflammatory response (22). It has been reported that the expression of ANGPTL8 is closely related to the plasma levels of triglyceride (TG) and very low density lipoprotein (23). Our team also found that ANGPTL8 promoted the lipogenic differentiation of mesenchymal stem cells, leading to adipoectopic deposition (24). In addition, it has been demonstrated that the plasma ANGPTL8 concentration increases significantly in patients with inflammatory response syndrome. Inflammatory factors, especially TNF-α, can upregulate the expression of ANGPTL8, and ANGPTL8 acts as a negative feedback regulator in TNF-α-triggered NF-κB activation (25). The ANGPTL8 R59W variant could increase the activity of NF-κB p65 and the circulatory levels of TNF-α and IL-7 (26). LPS-induced liver injury is closely related to immune cell activation and the expression and release of various inflammatory factors. However, the expression and function of ANGPTL8 in LPS-induced liver injury are poorly understood.

In this study, the role and mechanism of ANGPTL8 in LPS-induced liver injury were analyzed. We established a mouse model of LPS-induced liver injury by intraperitoneal injection of LPS and found that knockout of *Angptl8* ameliorates liver lipid deposition, lipid peroxidation, and the inflammatory response by activating the PGC1α/PPARα pathway. Therefore, inhibition of ANGPTL8 expression can improve metabolic disorders and the prognosis of sepsis patients.

## Materials and Methods

### Mice

C57BL/6J wild-type (WT) mice (6–8 weeks old, 20–25 g body weight) were obtained from the Animal Experimental Center of Hubei University of Medicine. C57BL/6J *Angptl8*^−/−^(*Angptl8* KO) mice were generated through the CRISPR/Cas9 system, and F0 and F1 generation animals were obtained from the Laboratory Animal Center of Sun Yat-sen University. The *Angptl8* sgRNAs were designed, cloned and inserted into the pX330 plasmid, and the recombinant *Angptl8* sgRNA-pX330 plasmid and Cas9 mRNA were co-injected into one-cell embryos. Thus, transgenic mice of the F0 generation were obtained, and the genotypes of the mice were analyzed via DNA sequencing. The F1 generation *Angptl8*^−/+^ (*Angptl8* heterozygotes) mice were transferred to the SPF Animal Experimental Center of Hubei University of Medicine.

All mice were housed (two to five per cage) in a controlled specific pathogen-free environment (12 h light/day cycle, 23 ± 1°C, 60%–70% humidity). The experimental procedures involving the animals were approved by the Center's Experimental Animal Ethics Committee. The animals were strictly bred and maintained in compliance with the National Institutes of Health Guide for the Care and Use of Laboratory Animals.

### Study procedures

The WT and *Angptl8* KO mice were randomly divided into a control group and three model groups. Mice in the model group were intraperitoneally (i.p.) injected with LPS (*E. coli* O55:B5, 10 mg/kg) and sacrificed at 24, 48, or 72 h after injection. Mice in the control group were injected i.p. with physiological saline solution. All the mice were euthanized after anesthesia. Blood samples were collected in heparin anticoagulant tubes and plasma was separated for hematological testing. Liver tissue was harvested for further analysis.

### Cells and culture conditions

The HepG2 cell line was purchased from the American Type Culture Collection (ATCC, Manassas) and used in the present study. The cells were cultured in DMEM supplemented with 10% FBS and maintained in a 5% CO_2_ incubator at 37°C. Null alleles for *Angptl8* were constructed using the CRISPRv2-Cas9 system. For HepG2 cells, guide RNAs (gRNAs) were designed, synthesized, cloned, and inserted into the lenti-CRISPRv2 plasmid (Genloci Biotechnologies, China). To package lentivirus, each lenti-CRISPRv2 plasmid with other components (psPAX2, pMD2G) was transfected into HEK293T cells using Lipofectamine™ 3,000 (Invitrogen). Transfected cells were cultured in DMEM containing 5% FBS, 100 U/ml penicillin, and 100 μg/ml streptomycin. The lentiviral particles were concentrated from the culture media and filtered through 0.45 μm filters using a Lenti-X Concentrator (Clontech). Aliquots were stored at −80°C until use. A total of 1.0×10^5^ HepG2 cells were plated in each well of a six-well plate and infected with the lentivirus mixed with polybrene. The transfected cells were selected by adding 2.5 μg/ml puromycin to the medium for 4–6 days. A lentivirus with a scrambled sequence was used as a control.

To study lipid deposition and ANGPTL8 expression in liver parenchymal cells under inflammatory conditions, HepG2 cells were stimulated with LPS. During the logarithmic growth phase, the HepG2 cells were washed and challenged with LPS dissolved in phosphate-buffered saline (PBS) at concentrations of 2, 4, or 8 μg/ml for 24 h. To determine the optimal incubation time, the cells were incubated with LPS (4 μg/ml) for 6, 12, or 24 h, respectively. Untreated HepG2 cells maintained in the medium for 6 h, 12 h or 24 h were used as controls. To clarify the function of ANGPTL8 in lipid deposition, *ANGPTL8* knockdown (KD) HepG2 cells and control HepG2 cells were treated with LPS (4 μg/ml).

### Liver transcriptomic analysis

RNA microarray analysis of liver tissue from WT and *Angptl8* KO mice after LPS stimulation for 48 h was performed using the DNBSEQ platform. We used Bowtie2 to map the clean reads to the reference gene sequence (transcriptome) and then used RSEM to calculate the gene expression level of each sample. Differential gene expression between groups was calculated using the DEseq2 method. According to the results of differential gene detection, the R package pheatmap was used to perform hierarchical clustering analysis on the union set differential genes.

### Biochemical parameters

The liver function indices alanine aminotransferase (ALT) and aspartate aminotransferase (AST) were detected as previously described (27). The concentrations of malondialdehyde (MDA), TG, and FFAs in liver homogenates were measured using commercial kits according to the instructions. The levels of TNF-α, IL-1β, and IL-6 in the serum were measured by ELISA, and the expression of liver inflammatory factors was quantified by qRT-PCR.

### Histological analysis

Hematoxylin and eosin (H&E) staining was performed on paraffin-embedded liver sections. To calculate the presence and extent of liver damage, H&E images were analyzed by a trained hepatopathologist who was blinded to the identity of the samples according to the criteria described by Kleiner *et al.* (28).

### qRT-PCR

Total RNA was extracted from frozen liver tissue using TRIzol (R1000, LABLEAD). Subsequently, cDNA was synthesized using the Superscript II Kit for qRT‒PCR (F0202, LABLEAD). qRT‒PCR was conducted in a 10 μl reaction mixture containing 50 nM forward and reverse primers, 1×SYBR Green reaction mix (R0202, LABLEAD), and 3.2 nM template. The sequences of the primers used for quantitative real-time PCR were as follows: 5′-CCTGGCACCCAGCACAAT-3′ and 5′-GGGCCGGACTCGTCATAC-3′ for human *β-ACTIN*; 5′-GTGCTATGTTGCTCTAGACTTCG-3′ and 5′-ATGCCACAGGATTCCATACC-3′ for mouse *β-actin*; 5′-ACCACAGGATAAGTCACCGAGGAG-3′ and 5′-TCGGCGAGGATAGTTCTGGAAGC-3′ for human *PPARα*; 5′-GATGTCACAGAACGGCTTCCTCAG-3′ and 5′-ACGATGCTGTCCTCCTTGATGAAC-3′ for mouse *Pparα*; 5′-TGATTTCTCCAGCATTTC-3′ and 5′-TGATCGCACTTTGGTATT-3′ for mouse *Pparγ*; 5′-CTGTCCGTGTTGTGTCAGGTCTG-3′ and 5′-GGGAGAGGCAGAGGCAGAAGG-3′ for human *PGC1α*; 5′-TTCCTCGTGTCCTCGGCTGAG-3′ and 5′-GTGCCACCGCCAACCAAGAG-3′ for mouse *Pgc1α*; 5′-GACGTGGAACTGGCAGAAGAG-3′ and 5′- TTGGTGGTTTGTGAGTGTGAG-3′ for mouse *Tnf-α*; 5′-TTCCCATTAGACAACTGC-3′ and 5′-GATTCTTTCCTTTGAGGC-3′ for mouse *Il-1β*. Fold changes were calculated using the delta–delta Ct method. Three biological replicates were performed for all the experiments.

### Western blot analysis

Liver tissues and HepG2 cells were collected and lysed in a lysis buffer (Beyotime Biotechnology). The proteins were extracted and separated by SDS‒PAGE and subsequently transferred onto PVDF membranes. After blocking with 5% BSA for 1 h at room temperature (RT), the membranes were incubated with antibodies against PPARα (sc-398394, Santa Cruz), PPARγ (16643-1-AP, Proteintech), PGC1α (ab54481, Abcam), and β-actin (AF7018, Affinity) at 4°C overnight. After washing 3 times with TBST, the membranes were incubated with horseradish peroxidase (HRP)-conjugated secondary antibody (A0216 or A0208, Beyotime Biotechnology) at RT for 1 h. The membranes were subsequently washed 3 times with TBST and imaged with a gel imaging system (Bio-Rad).

### Oil red O staining

The 0.5% Oil Red O stock solution was prepared with isopropanol and diluted to 0.3% using 60% isopropanol. The HepG2 cells were washed twice with PBS and fixed with 4% paraformaldehyde for 30 min. Subsequently, the cells were washed twice with double distilled water and soaked in 60% isopropanol for 30 s. The isopropanol was discarded, and the cells were incubated in Oil Red O working solution at 37°C for 20 min. The cells were washed once with isopropanol, followed by washing with double distilled water until the water became clear. The formation of lipid droplets (LDs) was observed using a bright field microscope (Olympus IX70) and Spot Advanced software. Frozen slices of liver tissue were prepared and then fixed with 4% paraformaldehyde. The Oil Red O staining of the slices followed the cell staining procedure.

### Statistical analysis

All the results are presented as the means ± -SEM or as dot plots. Unpaired two-tailed Student tests were used to compare the differences between the two groups. One-way ANOVA was used for comparisons among the different groups. The difference in mortality between groups was compared using the Kaplan‒Meier method. All the statistical analyses were performed with SPSS (version 19.0) and GraphPad (Version 8.0). A *P* value less than 0.05 was considered to indicate statistical significance.

## Results

### LPS causes ectopic lipid accumulation and lipotoxicity in the liver

**Fig. 1:**
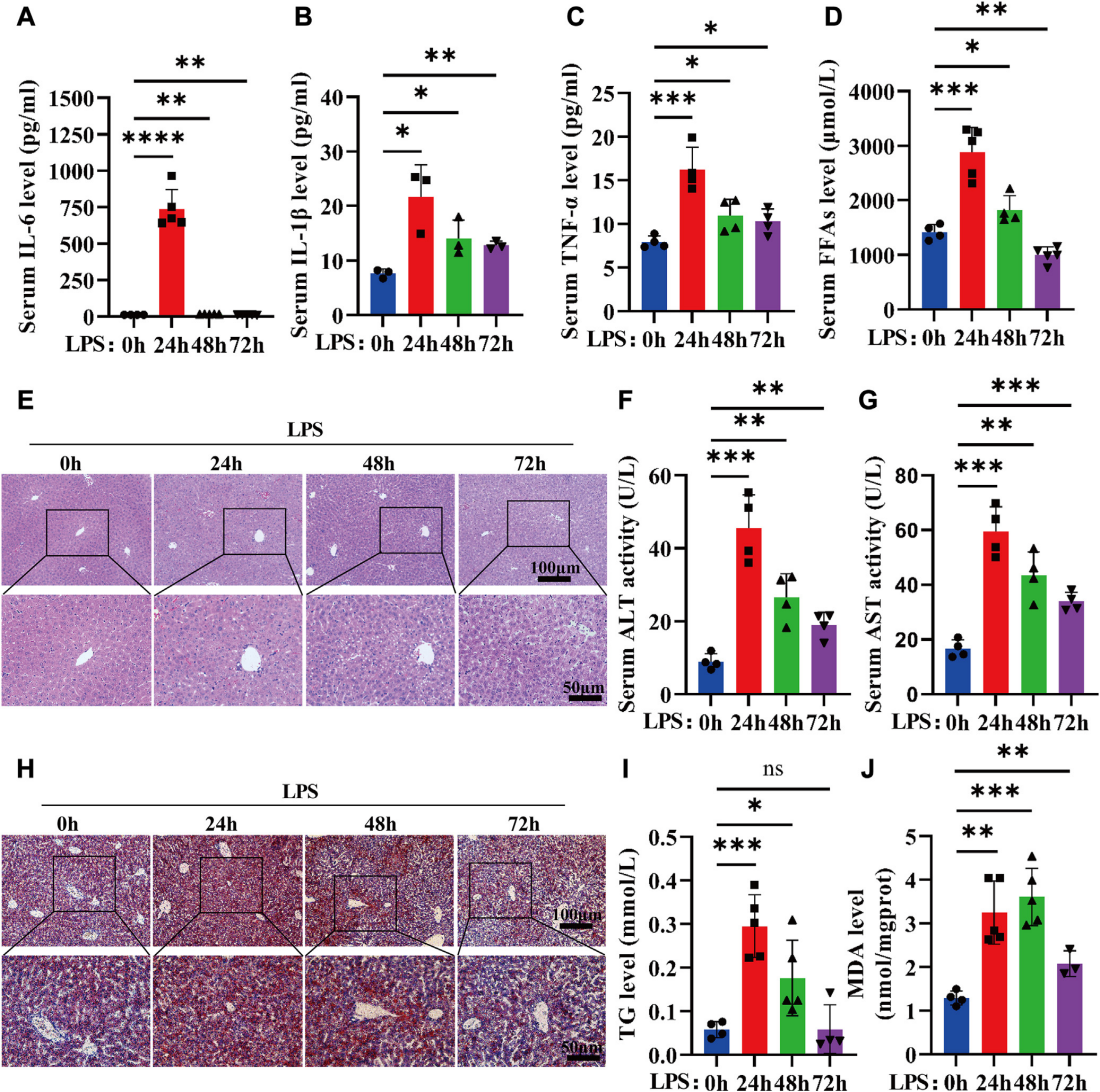
Increased lipid deposition and lipid peroxidation in the liver after LPS stimulation. A–C: Serum IL-6 (A), IL-1β (B) and TNF-α (C) levels after LPS (10 mg/kg) stimulation for 0 h, 24 h, 48 h, or 72 h (n = 3∼5 per group) were detected using ELISA kits. D: Serum FFAs levels after LPS (10 mg/kg) stimulation for 0 h, 24 h, 48 h, or 72 h (n = 5 per group) were detected using NEFA assay kits. E: H&E staining (A) of liver tissue after LPS (10 mg/kg) stimulation for 0 h, 24 h, 48 h, or 72 h (n = 5 per group). F and G: Serum alanine transaminase (ALT) (F) and aspartate transaminase (AST) (G) levels after LPS stimulation for 0 h, 24 h, 48 h, or 72 h (n = 4 per group) were measured via ALT and AST assay kits. H: Oil Red O staining of liver tissue after LPS (10 mg/kg) stimulation for 0 h, 24 h, 48 h, or 72 h (n = 5 per group). I and J: TG levels (I) and MDA levels (J) in liver homogenates from LPS-induced septic mice were detected using TG and MDA detection kits (n = 3∼5 per group). The data are shown as the mean ± SEM and were analyzed using an unpaired two-tailed Student’s test (C–F); ∗*P* < 0.05, ∗∗*P* < 0.01, ∗∗∗*P* < 0.001, ∗∗∗∗*P* < 0.0001, ns > 0.05.

Multiple studies have shown that sepsis can initiate the classic hunger response, which leads to adipose tissue lipolysis and the release of FFAs into the bloodstream (29). Excessive FFAs are mainly taken up by the liver and undergo fatty acid oxidation to provide energy for the body. However, infection can cause disturbances in liver fatty acid metabolism, which may lead to liver lipid deposition and peroxidation. In this study, we first analyzed the levels of inflammatory factors and FFAs in the serum of LPS-induced septic mice, and the results showed that LPS stimulation significantly increased the levels of IL-6, IL-1β, TNF-α, and FFAs (Fig. 1A–D). H&E staining revealed that the liver was characterized mainly by water degeneration and inflammatory cell infiltration at the early stage after LPS stimulation, and lipodroplet vacuoles were observed 48 h after LPS challenge (Fig. 1E). The plasma AST and ALT levels in the mice with sepsis were also significantly greater than those in the control mice (Fig. 1F, G). Lipid deposition in the liver was also assessed by Oil Red O staining and liver homogenate TG assays, which showed that lipid deposition in the liver was significantly increased 24 h after LPS challenge (Fig. 1H, I). It has been demonstrated that the key genes involved in β-oxidation in animal models of sepsis are inactivated in the liver, which results in the disruption of the hunger response and lipid peroxidation (30). We then detected the levels of MDA, which is one of the end products of lipid peroxidation in the liver. After 24 h of LPS stimulation, the MDA level in the liver homogenate increased nearly 3-fold, and remained higher than that in the control group until 72 h. (Fig. 1J). These results suggest that ectopic lipid deposition in the livers of LPS induced septic mice leads to lipid peroxidation, which may cause lipotoxicity and aggravate liver injury.

### ANGPTL8 is positively correlated with liver injury in patients with sepsis

**Fig. 2:**
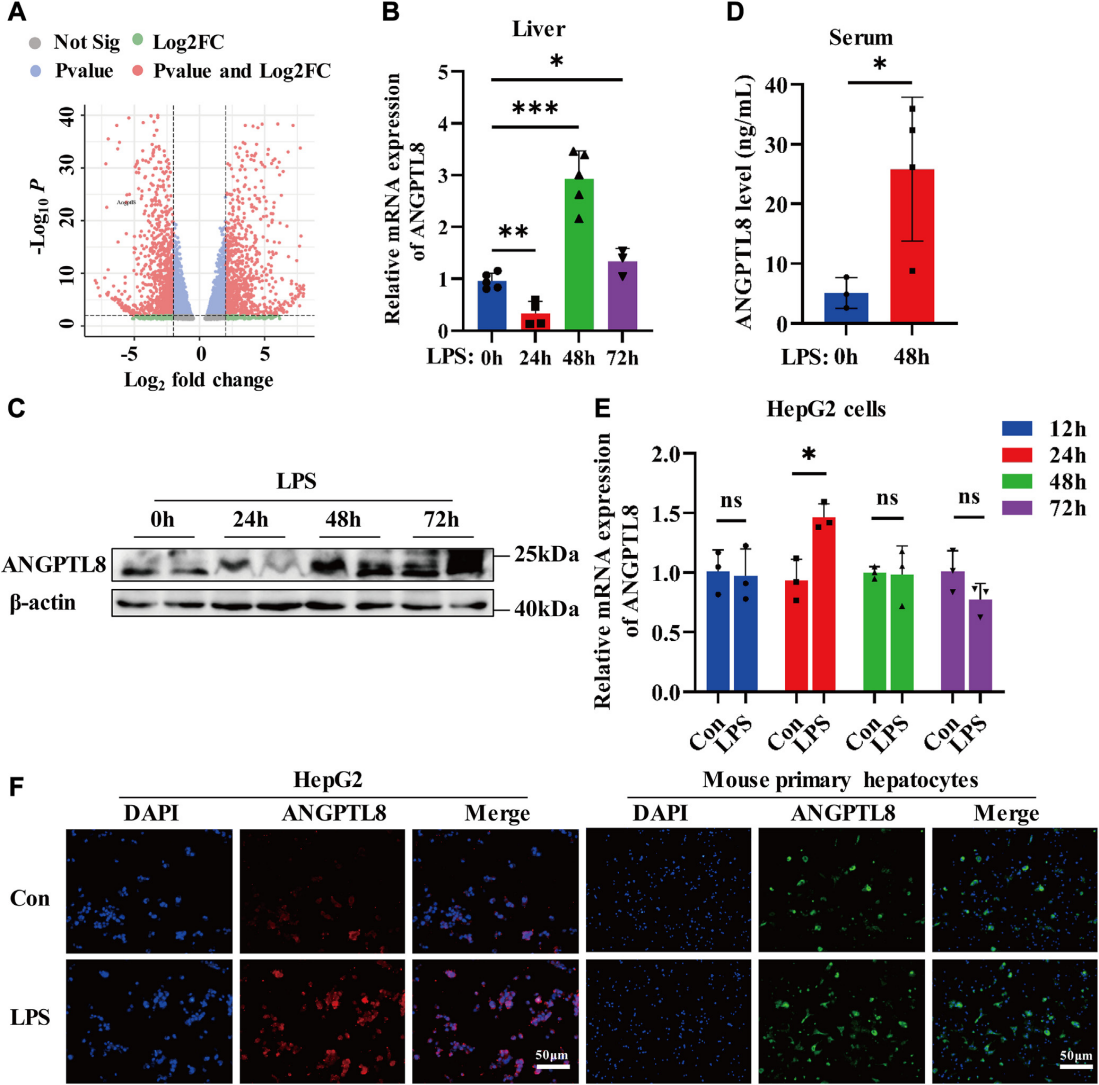
LPS upregulates the expression of ANGPTL8 in vivo and in vitro. A: The transcription level of ANGPTL8 in mice with sepsis was analyzed via the GEO database (GSE184167). B and C: the expression of ANGPTL8 in mouse liver tissue after LPS (10 mg/kg) stimulation for 0 h, 24 h, 48 h, or 72 h was detected via qRT‒PCR and Western blot (n = 3∼5 per group). D: serum ANGPTL8 levels after LPS stimulation for 48 h were measured using an ANGPTL8 ELISA kit (n = 3∼4 per group). E: qRT‒PCR analysis of the mRNA expression of ANGPTL8 in HepG2 cells after LPS (4 μg/ml) stimulation for 0 h, 12 h, 24 h, 48 h, or 72 h (n = 3 per group). F: the protein expression of ANGPTL8 in HepG2 cells and mouse primary hepatocytes after LPS treatment for 24 h or 4 h was determined by immunofluorescence (n = 3 per group). The data are shown as the mean ± SEM and were analyzed using an unpaired two-tailed Student’s test (B, D, E); ∗*P* < 0.05, ∗∗*P* < 0.01, ∗∗∗*P* < 0.001, ns > 0.05.

To clarify the mechanism of hepatic lipid peroxidation during sepsis, transcriptome sequencing data from the livers of septic mice from the GEO database (GSE224012) were analyzed. We found that the expression of ANGPTL8 in the livers of septic mice was significantly lower than that in the control group (Fig. 2A). ANGPTL8, which is expressed and secreted mainly by liver and adipose tissue, is closely associated with metabolic syndrome (24). To investigate the relationship between ANGPTL8 expression and lipid metabolism disorders in the liver during sepsis, we first detected the expression of ANGPTL8 in the liver and the plasma ANGPTL8 concentration in septic mice and control mice. As shown in Fig. 2B, C, the mRNA expression of ANGPTL8 in the liver was significantly inhibited at 24 h after LPS stimulation, but increased nearly 3-fold at 48 h after LPS stimulation. Consistent with the qRT‒PCR and Western blot results, the ELISA results also proved that plasma ANGPTL8 levels increased significantly at 48 h after LPS stimulation (Fig. 2D).

In addition, we stimulated HepG2 cells and primary hepatocytes with LPS and analyzed the expression of ANGPTL8 through qRT-PCR and immunofluorescence. The mRNA expression of ANGPTL8 in HepG2 cells was upregulated by LPS challenge, and the protein expression of ANGPTL8 in both HepG2 cells and primary hepatocytes was significantly elevated after LPS stimulation (Fig. 2E, F). Taken together, these data indicate that LPS can upregulate the expression of ANGPTL8 both in vivo and in vitro, suggesting that ANGPTL8 may participate in the pathological progression of liver injury caused by infection.

### ANGPTL8 deficiency reduces LPS-induced lipid deposition and peroxidation

**Fig. 3:**
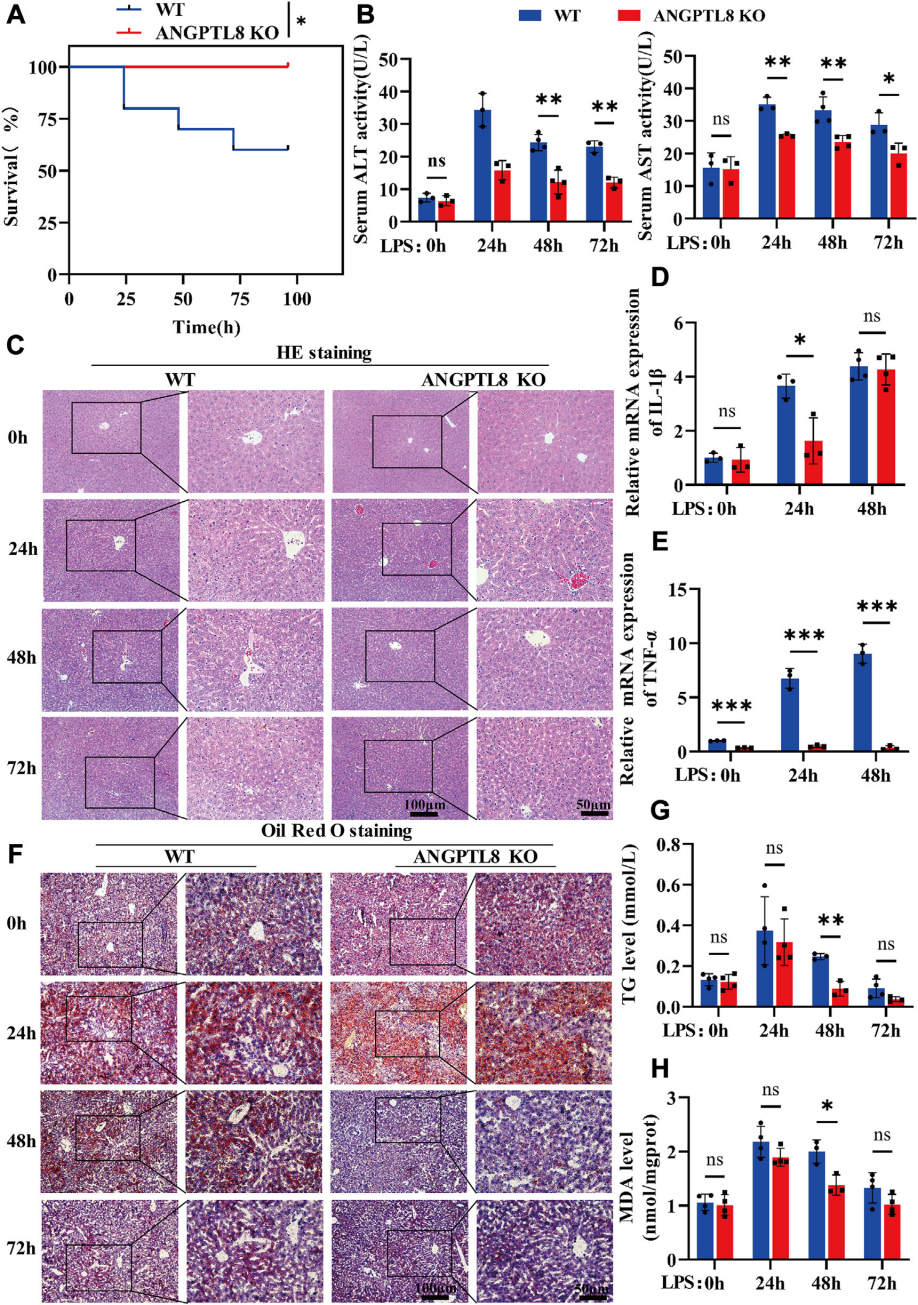
Knockout of *Angptl8* improves LPS-induced lipid deposition and peroxidation in the liver. A: The survival of *Angptl8* KO and WT septic mice induced by LPS (n = 10 per group). B: The serum ALT and AST levels after LPS stimulation for 0 h, 24 h and 48 h were measured via ALT and AST assay kits (n = 3∼4 per group). C: H&E staining was performed on the liver tissue of *Angptl8* KO and WT mice after LPS (10 mg/kg) stimulation for 0 h, 24 h, 48 h, or 72 h (n = 3∼4 per group). D and E: IL-1β (D) and TNF-α (E) expression in the livers of *Angptl8* KO and WT mice after LPS stimulation for 0 h, 24 h, and 48 h (n = 3∼4 per group) was detected and analyzed by qRT‒PCR. F: Oil Red O staining was performed on the liver tissue of *Angptl8* KO and WT mice after LPS (10 mg/kg) stimulation for 0 h, 24 h, 48 h, or 72 h (n = 3∼4 per group). G and H: TG levels (G) and MDA levels (H) in the liver homogenates of *Angptl8* KO and WT mice after LPS treatment were detected using TG and MDA detection kits (n = 3∼4 per group). The data are shown as the mean ± SEM and were analyzed using an unpaired two-tailed Student’s test (A, B, D, E, G, H); ∗*P* < 0.05, ∗∗*P* < 0.01, ∗∗∗*P* < 0.001, ns > 0.05.

**Supplemental Figure S1:**
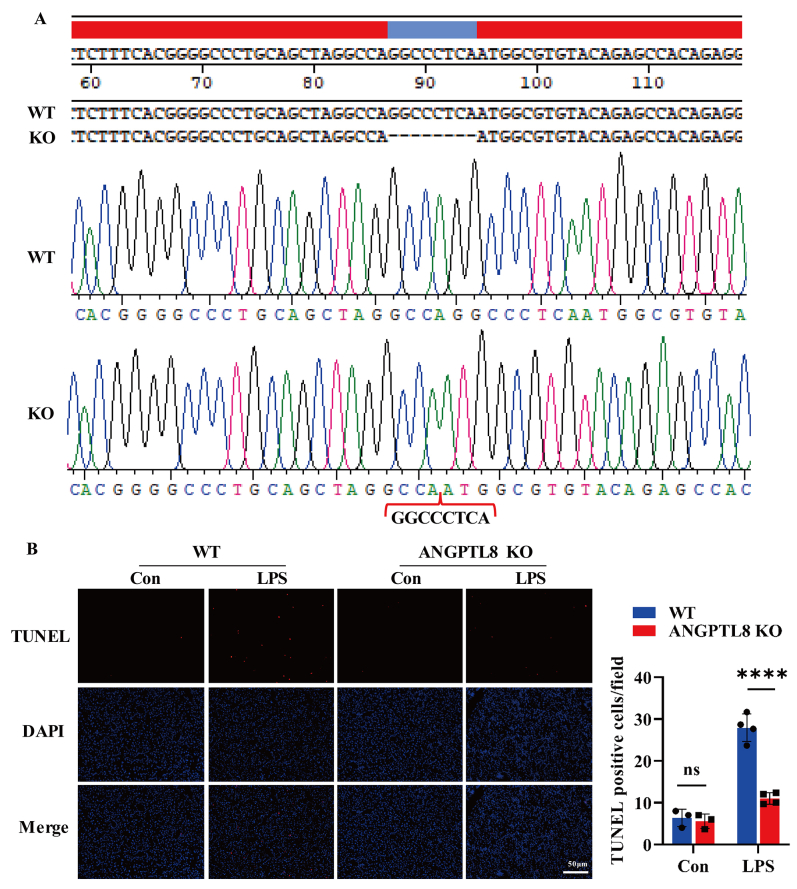
ANGPTL8 deficiency protects against LPS-induced apoptosis in liver tissue. A: The first exon of the ANGPTL8 gene in *Angptl8* KO mice is missing 8 bases, and the *Angptl8* KO mice were identified by DNA sequencing. B: TUNEL staining was used to analyse the number of apoptotic cells in liver tissue from *Angptl8* KO and WT mice after LPS (10 mg/kg) stimulation for 48 h (n = 3 per group). The data are shown as the mean ± SEM and were analysed using an unpaired two-tailed Student’s test ∗∗∗∗*p* < 0.0001, ns > 0.05.

To further elucidate the role of ANGPTL8 in LPS-induced liver injury, we constructed *Angpt8* KO mice and control mice (31), and the knockout of *Angptl8* was verified by sequencing (Supplemental Fig. S1A). Consistent with our hypothesis, genetic *Angptl8* depletion reduced the mortality rate of mice with LPS-induced sepsis (Fig. 3A), which might be attributed to improved liver function (Fig. 3B). Moreover, *Angptl8* KO reduced the infiltration of inflammatory cells into liver tissue (Fig. 3C), thereby reducing the expression of inflammatory factors such as IL-1β and TNF-α (Fig. 3D, E). HE staining also revealed a decrease in the number of adipose vacuoles in the liver tissue of *Angptl8* KO mice and WT mice. We further investigated the difference in lipid deposition in the livers of *Angptl8* KO mice and WT mice. As expected, the number of LD in the livers of *Angptl8* KO mice was significantly lower than that in the livers of WT mice at 48 h after LPS stimulation (Fig. 3F). The TG content in the liver homogenate of *Angptl8* KO mice was also lower than that in the livers of WT mice (Fig. 3G). Ectopic lipid deposition is closely related to lipid peroxidation, which further exacerbates tissue damage. We analyzed the levels of MDA, a lipid peroxidation product, in liver tissue homogenates. As shown in Fig. 3J, the level of MDA in the liver tissue of *Angptl8* KO mice was significantly lower than that in the livers of WT mice (Fig. 3H). Subsequently, we performed TUNEL staining on liver tissue from mice stimulated with LPS for 48 h. Compared with those in the livers of the WT mice, the number of apoptotic cells in the livers of *Angptl8* KO mice was markedly reduced (Supplemental Fig. S1B).

**Supplemental Figure S2:**
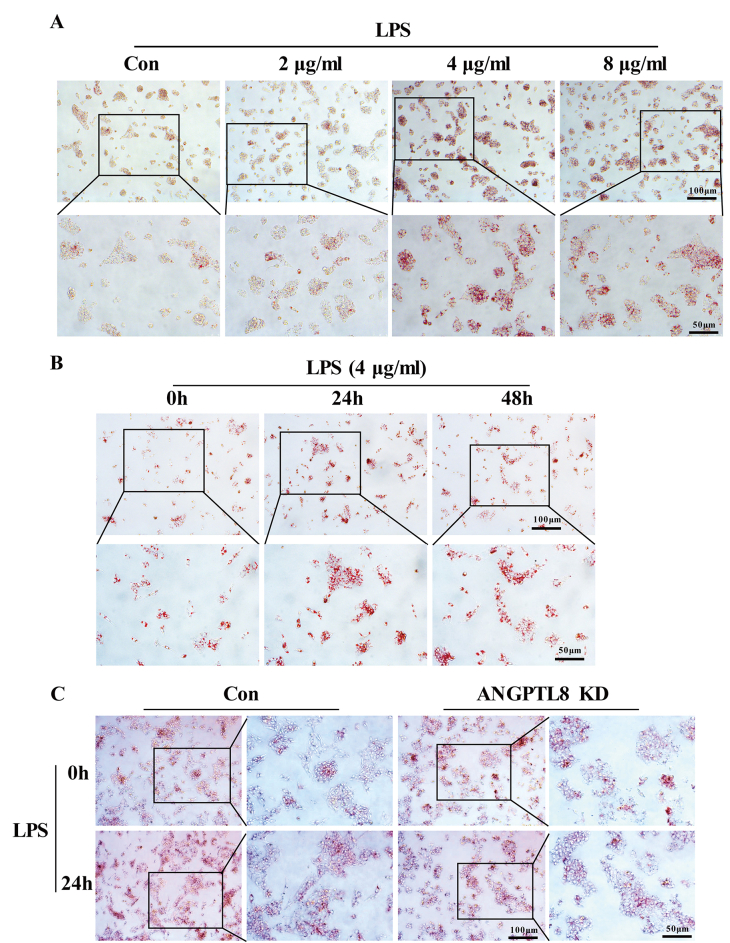
LPS promotes lipid deposition in HepG2 cells. A: Oil Red O staining analysis of lipid deposition in HepG2 cells after LPS (2 μg/ml, 4 μg/ml or 8 μg/ml) stimulation for 24 h. B: Lipid deposition in HepG2 cells was detected by Oil Red O staining after LPS (4 μg/ml) stimulation for 24 h or 48 h. C: Lipid deposition in *ANGPTL8* KD HepG2 cells and control HepG2 cells after LPS (4 μg/ml) stimulation for 24 h was detected by Oil Red O staining.

We constructed an *ANGPTL8* KD HepG2 cell line as previously described (32) and explored the role of ANGPTL8 in lipid metabolism during LPS stimulation. We found that the number of lipid droplets in HepG2 cells increased significantly after LPS (4 μg/ml) stimulation for 24 h (Supplemental Fig. S2A, B). However, the formation of lipid droplets in *ANGPTL8* KD cells did not change significantly after LPS treatment (Supplemental Fig. S2C). Overall, we demonstrated that the inhibition of ANGPTL8 reduces LPS-induced lipid accumulation both in vivo and in vitro.

### ANGPTL8 deficiency activates the PGC1α/PPARα pathway

**Fig. 4:**
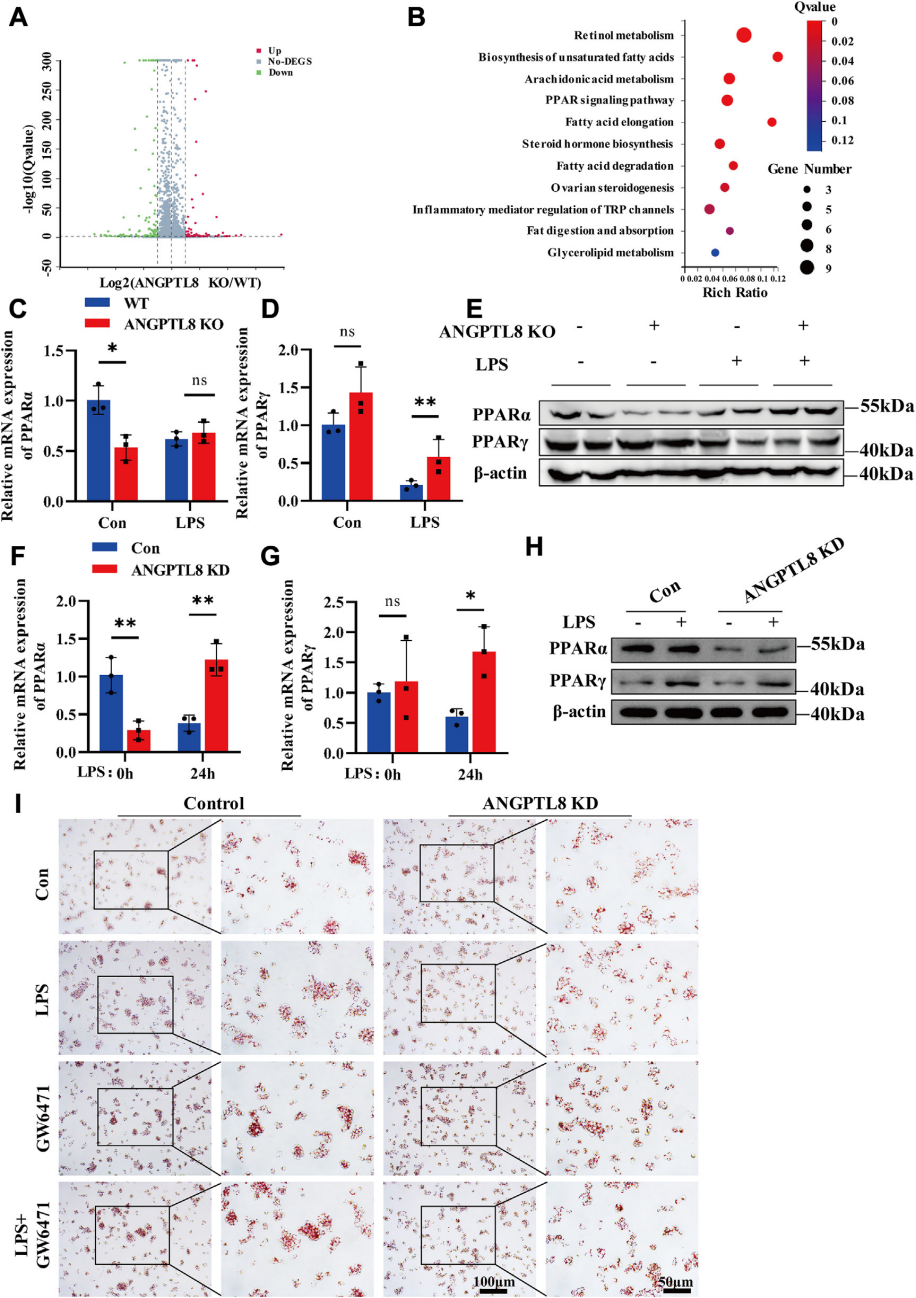
ANGPTL8 deficiency potentiates liver PPARα expression. A: The above results demonstrated that the lipid deposition and peroxidation indices in the livers of *Angptl8* KO mice and control mice were significantly different after LPS stimulation for 48 h. Volcano plot analysis of the RNA sequences from the livers of *Angptl8* KO and WT mice after LPS stimulation for 48 h is shown (n = 3 per group). B: KEGG enrichment analysis of the *Angptl8* KO and WT groups to predict the functions of ANGPTL8 during LPS-induced liver injury (n = 3 per group). C and D: The transcriptional levels of PPARα (C) and PPARγ (D) in the liver tissue of *Angptl8* KO and WT mice after LPS (10 mg/kg) stimulation for 48 h (n = 3∼4 per group). E: The protein levels of PPARα and PPARγ in the liver tissue of *Angptl8* KO and WT mice after LPS (10 mg/kg) stimulation for 48 h (n = 3 per group). F and G: qRT‒PCR analysis of the mRNA expression of PPARα (G) and PPARγ (H) in control HepG2 cells and *ANGPTL8* KD HepG2 cells after LPS (4 μg/ml) stimulation for 24 h. H: Western blot analysis of the protein levels of PPARα and PPARγ in *ANGPTL8* KD HepG2 cells and control HepG2 cells after LPS (4 μg/ml) stimulation for 24 h. I: *ANGPTL8* KD HepG2 cells and control HepG2 cells were first stimulated with LPS (4 μg/ml) for 24 h, and then treated with PPARα inhibitor GW6471 (10 μM) for 24 h, and lipid deposition was analysed by Oil Red O staining. The data are shown as the mean ± SEM and were analysed using an unpaired two-tailed Student’s test (C, D, F, G); ∗*P* < 0.05, ∗∗*P* < 0.01, ns > 0.05.

Previous studies have shown that ANGPTL8 regulates lipoprotein lipase activity, which facilitates TG utilization by peripheral tissues under fasting conditions and promotes lipid storage under feeding conditions (33, 34). Our previous studies revealed that ANGPTL8 promoted high-fat diet-induced liver inflammation and fibrosis through the LILRB2/ERK pathway (32). To elucidate the mechanism by which ANGPTL8 regulates liver lipid metabolism during sepsis, we performed RNA-seq analysis of livers from WT and ANGPTL8 KO mice 48 h after LPS stimulation. A volcano plot was constructed to show the differential gene expression (>2-fold) profiles of the WT and *Angptl8* KO mice (Fig. 4A). Subsequently, we performed Kyoto Encyclopedia of Genes and Genomes (KEGG) analysis to determine the functions of these differentially expressed genes (DEGs). The results showed that the DEGs were mainly involved in fatty acid metabolism, including the biosynthesis of unsaturated fatty acids, the PPAR signaling pathway, and fatty acid degradation (Fig. 4B). PPARs, including PPARα, PPARγ, and PPARβ/δ, are fatty acid-activated transcription factors that participate in regulating fatty acid metabolism, energy balance and the inflammatory response (35, 36). Many studies have shown abnormal expression of PPARα and PPARγ in the pathological state of sepsis, which is closely related to patient prognosis (21, 37). Under physiological conditions, *Angptl8* KO significantly reduced liver PPARα expression. However, there was no difference in the mRNA expression of PPARα between *Angptl8* KO mice and WT mice, and the protein expression in *Angptl8* KO mice was greater than that in WT mice after LPS stimulation (Fig. 4C, E). We also analyzed the repression of PPARγ, and the results showed that there was no difference in the liver expression of PPARγ between ANGPTL8 KO mice and WT mice without LPS stimulation. However, under LPS stimulation, the mRNA expression of PPARγ in the livers of *Angptl8* KO mice was greater than that in the livers of WT mice, but the protein expression of PPARγ did not significantly change (Fig. 4D, E). Next, we examined whether knockdown of *ANGPTL8* can ameliorate lipid deposition through PPARα. After LPS challenge, PPARα expression was considerably decreased in control HepG2 cells and significantly increased in *ANGPTL8* KD HepG2 cells (Fig. 4F, H). However, there was no significant difference in PPARγ mRNA expression in either control HepG2 cells or *ANGPTL8* KD HepG2 cells after LPS challenge, but the protein level of PPARγ increased after LPS challenge in both control HepG2 cells and *ANGPTL8* KD HepG2 cells (Fig. 4G, H). To determine whether the regulation of lipid deposition by ANGPTL8 is dependent on PPARα, the PPARα inhibitor GW6471 was used to treat control HepG2 cells and *ANGPTL8* KD HepG2 cells. The Oil Red O staining results showed that inhibiting PPARα reversed the alleviating effect of *ANGPTL8* KD on LPS-induced lipid deposition (Fig. 4I). Taken together, these results suggest that knocking out *Angptl8* can improve liver lipid metabolism by upregulating PPARα expression.

**Fig. 5:**
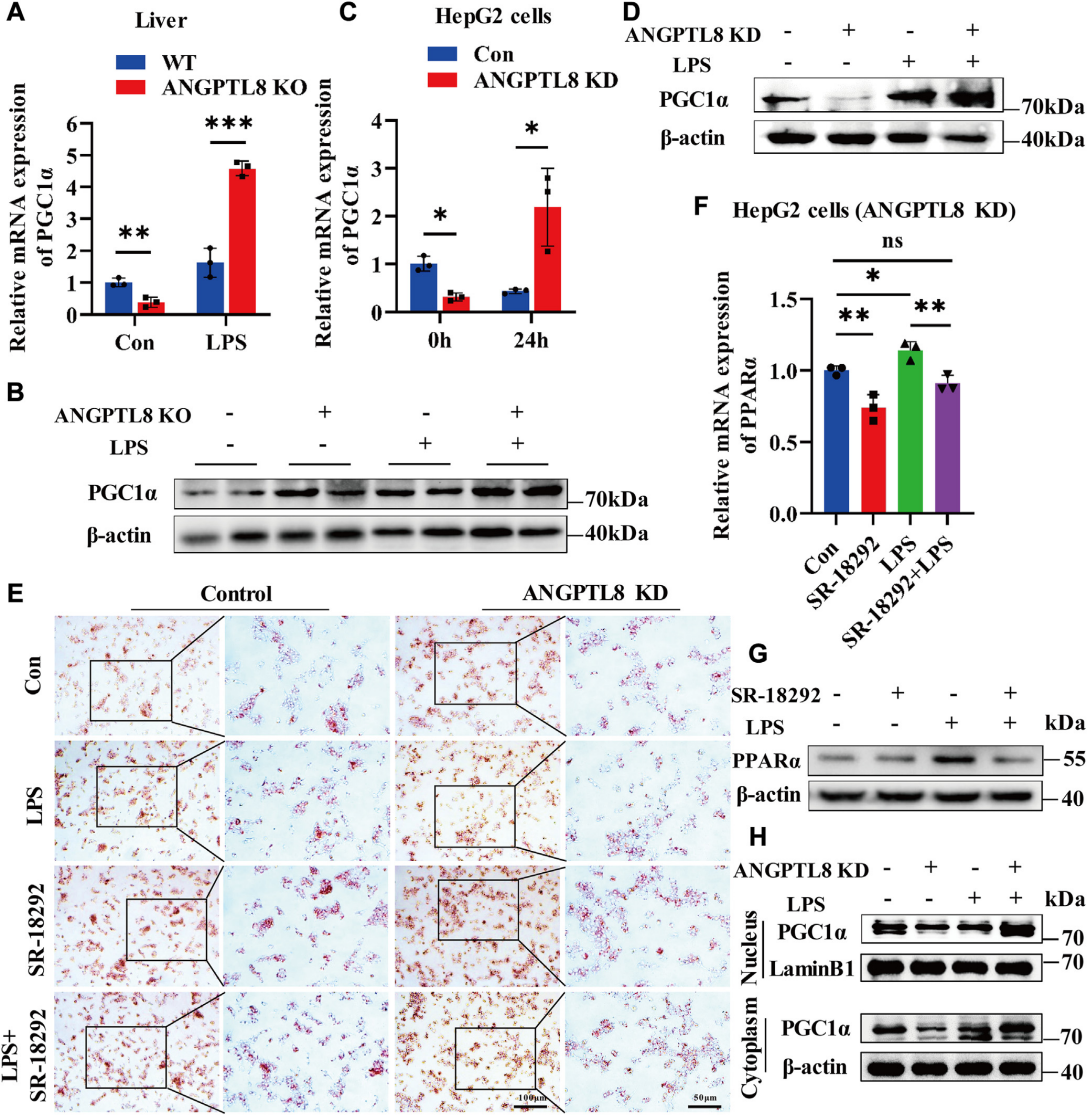
The LPS-induced upregulation of PPARα depends on PGC1α. A and B: qRT‒PCR and Western blot analysis of the mRNA (A) and protein (B) expression of PGC1α in the liver tissue of *Angptl8* KO and WT mice after LPS (10 mg/kg) stimulation for 48 h (n = 3∼5 per group). C and D: qRT‒PCR and Western blot analysis of the mRNA (C) and protein (D) expression of PGC1α in *ANGPTL8* KD HepG2 cells and control HepG2 cells after LPS (4 μg/ml) stimulation for 24 h. E: Oil Red O staining analysis of lipid deposition in *ANGPTL8* KD HepG2 cells and control HepG2 cells after stimulation with the PGC1α inhibitor SR-18292 (20 μM) and LPS for 24 h. F and G: qRT‒PCR and Western blot analysis of the mRNA (F) and protein (G) expression of PPARα in *ANGPTL8* KD HepG2 cells after stimulation with SR-18292 and LPS for 24 h. H: Western blot analysis of the protein level of PGC1α in the cytoplasm and nucleus of *ANGPTL8* KD HepG2 cells and control HepG2 cells after LPS (4 μg/ml) stimulation for 24 h; β-actin and lamin B1 were used as reference for the cytoplasm and nucleus, respectively. The data are shown as the mean ± SEM and were analysed using an unpaired two-tailed Student’s test (A, C, F), ∗*P* < 0.05, ∗∗*P* < 0.01, ∗∗∗*P* < 0.001, ns > 0.05.

**Supplemental Figure S3:**
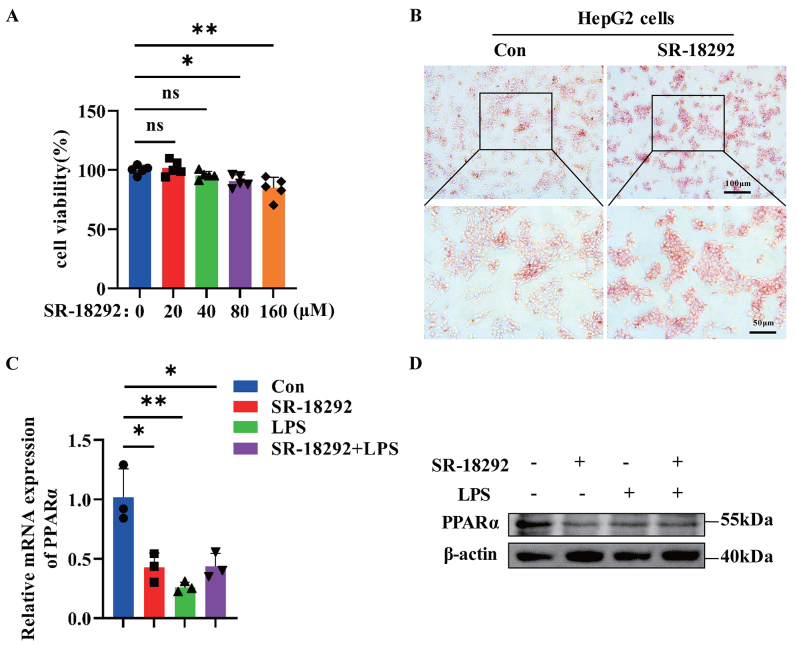
The inhibitory effects of LPS on PPARα were dependent on PGC1α. A: The optimal treatment duration and concentration of the PGC1α inhibitor SR-18292 were determined via a CCK8 assay. B: Oil Red O staining analysis of lipid deposition in HepG2 cells after SR-18292 (20 μM) treatment for 24 h. C and D: The expression of PPARα in HepG2 cells after SR-18292 treatment was analysed through qRT‒PCR (C) and Western blot (D). The data are shown as the mean ± SEM and were analysed using an unpaired two-tailed Student’s test; ∗*p* < 0.05, ∗∗*p* < 0.01, ns > 0.05.

The transcription factor peroxisome-proliferator-activated receptor coactivator 1 alpha (PGC1α) plays an important role in mitochondrial biogenesis and metabolism (38). We found that the suppression of PGC1α by the PGC1α inhibitor SR18292 in HepG2 cells promoted lipid deposition (Supplemental Fig. S3A, B). The expression of peroxisome proliferator-activated receptor coactivator (PGC1α) in the livers of *Angptl8* KO and WT septic mice was also analyzed. Compared with that in the livers of WT control mice, the mRNA expression of PGC1α in the livers of WT septic mice increased less than 1-fold, while the expression of PGC1α in the *Angptl8* KO mice increased more than 9-fold compared to that in the *Angptl8* KO control mice (Fig. 5A). Consistent with the qRT-PCR results, the protein expression of PGC1α was also upregulated in the livers of *Angptl8* KO mice (Fig. 5B). However, after LPS stimulation in vitro, PGC1α expression was decreased in control HepG2 cells and significantly increased in *ANGPTL8* KD HepG2 cells (Fig. 5C, D). Then, the PGC1α inhibitor SR-18292 was applied to clarify whether the regulation of PPARα by ANGPTL8 depends on PGC1α. Notably, SR-18292 treatment promoted lipid deposition in both control HepG2 cells and *ANGPTL8* KD HepG2 cells, and the addition of SR-18292 increased LPS-induced lipid deposition in *ANGPTL8* KD HepG2 cells (Fig. 5E). Consistent with the Oil Red O results, the LPS-induced upregulation of PPARα expression in *ANGPTL8* KD HepG2 cells was significantly suppressed by SR-18292 (Fig. 5F, G), which was not found in control HepG2 cells (Supplemental Fig. S3C, D). Furthermore, after LPS stimulation, the protein level of PGC1α in the cytoplasm and nucleus was increased in both the cytoplasm and nucleus of *ANGPTL8* KD HepG2 cells (Fig. 5H), which indicated that PGC1α may directly regulate the expression of PPARα, a transcription factor. Overall, ANGPTL8 deficiency inhibits LPS-induced lipid deposition by activating the PGC1α/PPARα signaling pathway.

### Upregulation of ANGPTL8 expression induced by LPS depends on TNF-α

**Fig. 6:**
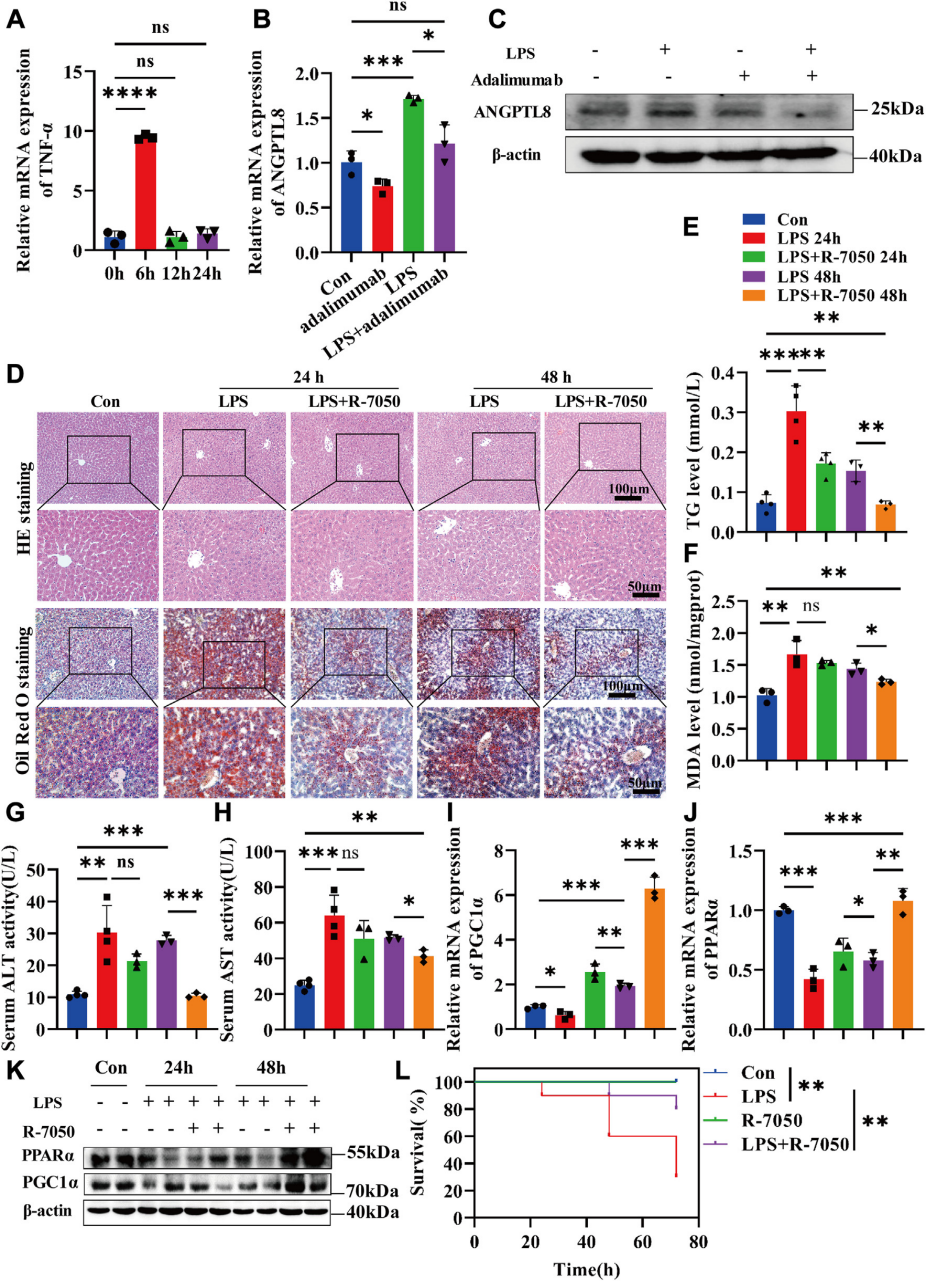
Inhibition of TNF-α can ameliorate LPS-induced lipid deposition in vivo and in vitro. A: qRT‒PCR detection of TNF-α mRNA expression at different time points after LPS (4 μg/ml) stimulation. B and C: The mRNA (B) and protein (C) expression of ANGPTL8 in HepG2 cells after LPS or adalimumab (5 μg/ml) treatment for 24 h was measured by qRT‒PCR and Western blot, respectively. D: H&E staining and Oil Red O staining analysis of pathology and lipid deposition in the livers of septic mice treated with the TNF-α receptor inhibitor R-7050 (6 mg/kg) for 24 h and 48 h, respectively (n = 3∼4 per group). E and F: TG levels (D) and MDA levels (E) in liver homogenates from septic mice after R-7050 treatment for 24 h or 48 h were measured (n = 3∼4 per group). G and H: The serum ALT (G) and AST (H) levels of septic mice after R-7050 treatment for 24 h or 48 h were detected (n = 3∼4 per group). I–K: The expression of PGC1α and PPARα in the livers of septic mice after R-7050 treatment for 24 h or 48 h was measured by qRT‒PCR (I and J) and Western blot (K) (n = 3 per group). L: The survival of septic mice after R-7050 treatment was observed and statistically analyzed. The data are shown as the mean ± SEM and were analyzed using an unpaired two-tailed Student’s test (A and B); ∗*P* < 0.05, ∗∗*P* < 0.01, ∗∗∗*P* < 0.001, ∗∗∗∗*P* < 0.0001, ns > 0.05.

**Supplemental Figure S4:**
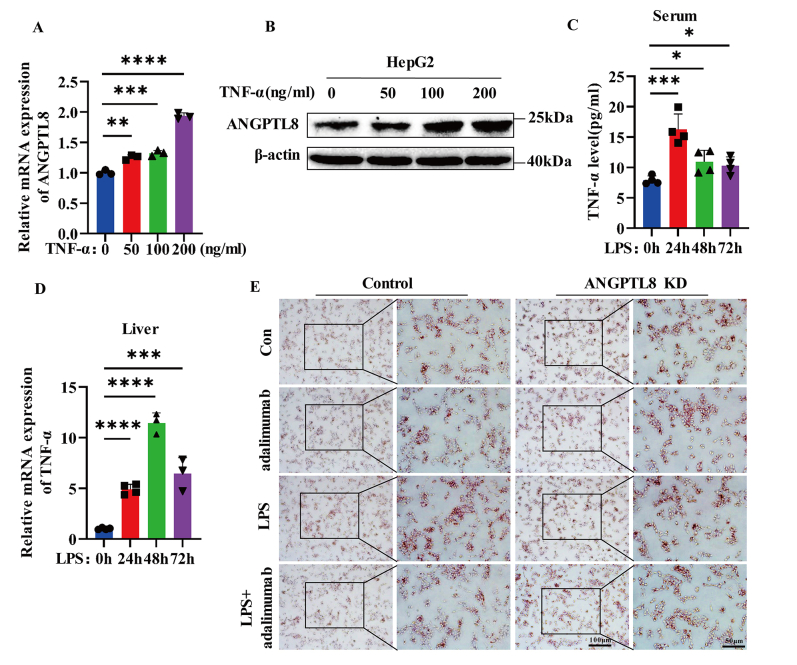
Regulation of lipid metabolism by ANGPTL8 during sepsis depends on TNF-α. A and B: HepG2 cells were stimulated with TNF-α at 50, 100 and 200 ng/ml, respectively, and ANGPTL8 expression was measured by qRT‒PCR (A) and Western blot (B). C: ELISA was used to measure the serum TNF**-**α concentration after LPS stimulation for 24 h, 48 h, or 72 h (n = 4 per group). D: The mRNA expression of TNF**-**α in liver tissue from LPS challenged mice was detected through qRT‒PCR (n ≥ 3 per group). E: Oil Red O staining analysis of lipid deposition in *ANGPTL8* KD HepG2 cells and control HepG2 cells after treatment with LPS (4 μg/ml) or the TNF-α monoclonal antibody adalimumab (5 μg/ml) for 24 h. The data are shown as the mean ± SEM and were analysed using an unpaired two-tailed Student’s test. ∗*p* < 0.05, ∗∗*p* < 0.01, ∗∗∗*p* < 0.001, ∗∗∗∗*p* < 0.0001.

During infection, excessive cytokine release and immune cell infiltration play important roles in lipid metabolism. Cytokines can promote fatty acid uptake in the liver, which may lead to lipid deposition in the liver (39). Moreover, immune cells can also participate in the regulation of lipid metabolism (40). Inflammatory stimuli have been shown to regulate ANGPTL8 expression (25). Our results also proved that TNF-α enhances ANGPTL8 expression in a concentration-dependent manner in HepG2 cells (Supplemental Fig. S4A, B). Subsequently, we determined the influence of LPS on TNF-α expression in HepG2 cells. As expected, the expression of TNF-α in HepG2 cells was significantly increased after LPS stimulation (Fig. 6A). Furthermore, the serum TNF-α concentration and TNF-α mRNA expression in the livers of LPS-treated mice were significantly increased (Supplemental Fig. S4C, D). To further clarify the effect of TNF-α on ANGPTL8, the TNF-α antibody adalimumab was used to block TNF-α. As shown in Fig. 6 (B, C), the LPS-induced upregulation of ANGPTL8 expression was inhibited by adalimumab. Notably, adalimumab alleviated LPS-induced lipid deposition in control HepG2 cells, but not in *ANGPTL8* KD HepG2 cells (Supplemental Fig. S4E).

**Fig. 7:**
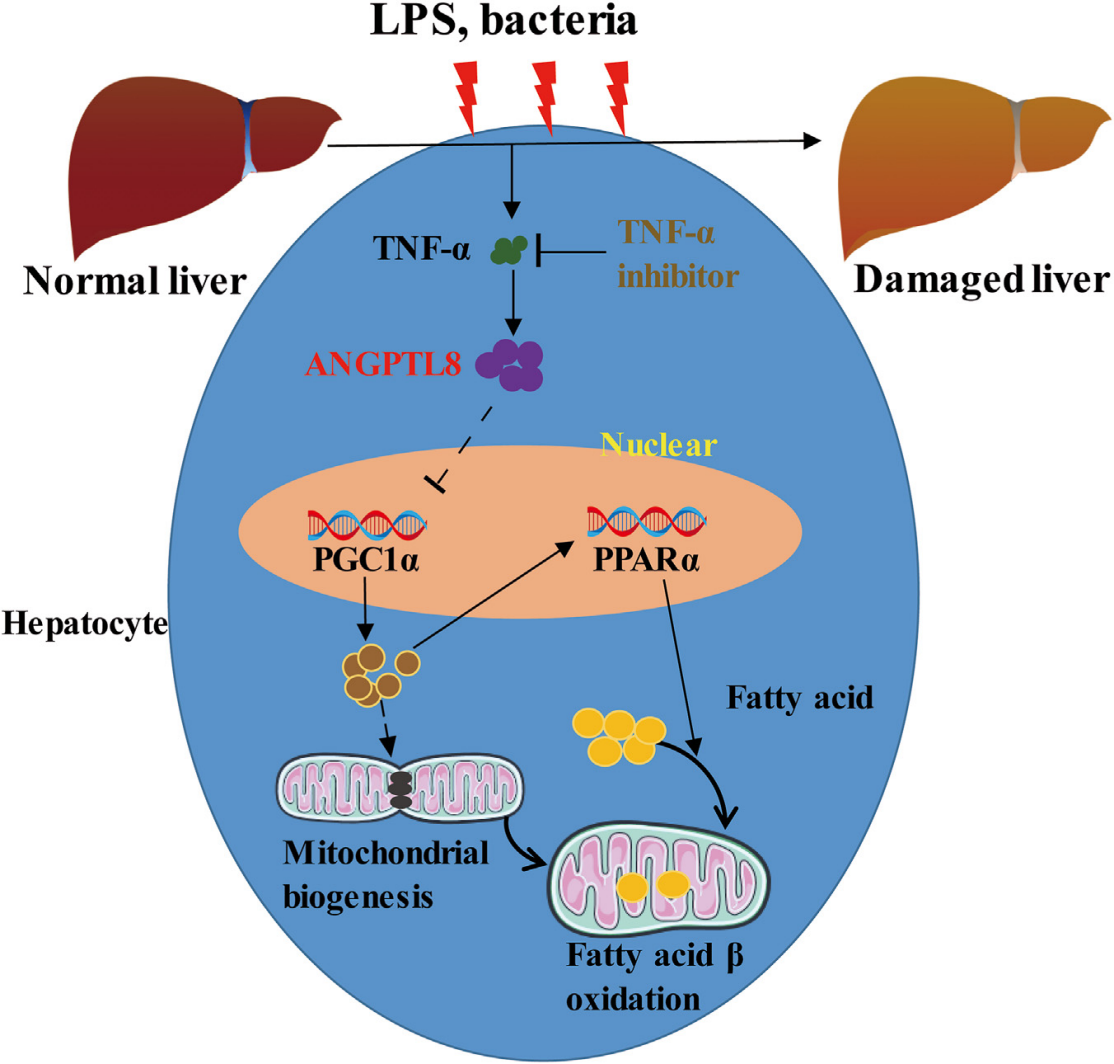
Working model of the ANGTPL8-mediated regulation of PGC1α/PPARα signaling. Infection upregulates liver TNF-α expression, which further promotes ANGPTL8 expression. ANGPTL8 can inhibit the expression and activation of PGC1α. The expression of the downstream gene of PPARα, was also downregulated, which blocks fatty acid β-oxidation and leads to lipid deposition and liver damage.

Furthermore, the TNF-α receptor antagonist R-7050 was used to treat LPS-stimulated mice. Liver lipid lesion formation, lipid deposition, and lipid peroxidation were significantly improved at 24 h and 48 h after R-7050 treatment (Fig. 6D–F). The levels of the serum liver function indices ALT and AST were significantly decreased after R-7050 treatment (Fig. 6G, H). The expression of PGC1α and PPARα in the liver was also detected, and the results showed that the inhibition of TNF-α can upregulate the expression of PGC1α and PPARα (Fig. 6I–K). Moreover, R-7050 significantly improved the survival rate of septic mice (Fig. 6L). Overall, inhibition of TNF-α can increase the survival rate of septic mice by improving liver ANGPTL8/PGC1α/PPARα-mediated lipid metabolism (Fig. 7).

## Discussion

Liver dysfunction, which is caused mainly by excessive inflammation and lipid peroxidation, is closely related to the prognosis of sepsis patients (41). Here, we demonstrated the potential role of ANGPTL8 in regulating hepatic ectopic lipid deposition and lipid peroxidation during LPS-induced inflammation. In response to the LPS challenge, ANGPTL8 expression was increased in liver tissue, and ANGPTL8 deficiency decreased lipid deposition and peroxidation by activating the PGC1α/PPARα signaling pathway. These findings establish ANGPTL8 as a potential therapeutic target for treating hepatic injury in patients with sepsis caused by bacterial infection.

Recent studies have shown significant changes in lipid and lipoprotein concentrations and composition in critically ill patients (42). Once infection occurs, the body may experience a series of acute reactions, such as fever, shortness of breath, and immune activation, all of which require an energy supply that exceeds normal physiological requirements. Therefore, sepsis can cause a strong hunger response that leads to lipolysis of adipose tissue (43). However, liver mitochondrial function and fatty acid β-oxidation are impaired during sepsis. Our results further demonstrated that severe liver lipid deposition and lipid peroxidation occur in the early stages of LPS stimulation. Lipid accumulation in the liver is usually considered a transient metabolic adaptation to starvation, and excess lipids are stored in the form of LDs to avoid toxic stimuli. In this study, we demonstrated that LPS stimulation not only promotes hepatic lipid accumulation and LDs formation but also increases lipid peroxidation, which increases the level of toxic lipid peroxidation byproducts such as MDA. The accumulation of these lipid peroxidation byproducts can exacerbate inflammation, oxidative stress, and cell apoptosis, which eventually exacerbates liver damage and reduces the survival of patients with sepsis. Therefore, further elucidation of the mechanism of hepatic lipid metabolism disorders during sepsis will contribute to the development of new therapeutic options and improve the prognosis of sepsis patients.

ANGPTLs reportedly participate in a variety of critical cellular processes, such as angiogenesis, glucose metabolism, differentiation, and lipid metabolism (44). Studies have shown that ANGPTL8 and ANGPTL3 synergistically inhibit lipoprotein protease activity and increase peripheral blood TG content (45). Circulating ANGPTL8 levels are significantly increased in patients with nonalcoholic fatty liver disease and type 2 diabetes mellitus (46, 47). Our previous study revealed that ANGPTL8 promotes the lipogenic differentiation of mesenchymal stem cells and fat deposition in male mice (24). Here, we showed that the expression of ANGPTL8 in the livers of LPS-induced septic mice was significantly increased, which was consistent with the findings of previous reports (25). However, a significant decrease in the expression of ANGPTL8 24 h after LPS injection was observed in our study, which may be due to the metabolic response to starvation caused by acute infection. Further research showed that knocking out ANGPTL8 alleviated LPS-induced lipid accumulation, lipid peroxidation, and inflammation in the liver, thereby improving the survival of septic mice. Notably, in the early stage of the LPS challenge, liver lipid deposition increased dramatically in both *Angptl8* KO mice and WT mice, but there was no difference between the two groups. It is widely recognized that the FFAs and glycerol released by adipose tissue quickly enter the bloodstream when the body has an is infection. These FFAs are mainly absorbed by the liver and undergo fatty β-oxidation to produce energy and metabolic intermediates (48). However, fatty β-oxidation is blocked during severe sepsis, which leads to excessive lipid deposition and peroxidation. Therefore, we speculate that ANGPTL8 deficiency does not affect liver FFAs uptake but rather alleviates liver lipid deposition and injury by promoting fatty β-oxidation, thereby improving the prognosis of patients with sepsis and that the mechanism by which ANGPTL8 regulates fatty β-oxidation needs to be further elucidated.

As shown in previous studies, lipid metabolism is regulated by multiple genes, including SREBP1, PPARs, CD36, and AMPK (36, 41, 49, 50, 51). To clarify the mechanism by which ANGPTL8 regulates liver lipid metabolism during infection, we performed RNA-seq analysis of livers dissected from LPS-challenged WT mice and ANGPTL8 KO mice. The results demonstrated that some of the differentially expressed genes, including PPARα, were associated with fatty acid metabolism. As a kind of transcription factor, PPARα can stimulate the expression of genes involved in lipid metabolism through binding directly to the promoter regions of these genes (52). It has been proven that PPARα reduces plasma TG levels and liver lipid deposition by promoting the transfer of FFAs into mitochondria. Therefore, improving mitochondrial function can be beneficial for reducing lipid deposition. PGC1α, recognized as PPARγ coactivator 1 alpha, is one of the main regulators of mitochondrial biogenesis and metabolic processes (53). In the liver, PGC1α can also collaborate with PPARα to activate genes associated with fatty β-oxidation, which increases energy production (54). In this study, we demonstrated that the expression of PGC1α in the livers of *Angptl8* KO mice was significantly increased after LPS stimulation and that the downregulation of PPARα by ANGPTL8 depended on the inhibition of PGC1α. These results indicated that ANGPTL8 may participate in liver injury associated with sepsis through regulating mitochondrial metabolism and biogenesis. Future investigations will further clarify the mechanism by which ANGPTL8 regulates PGC1α.

The inflammatory response has been demonstrated to regulate ANGPTL8 expression (25). As one of the main inflammatory mediators, TNF-α plays an important role in multiple biological processes, including the inflammatory response, cell proliferation, and cell death. Hence, abnormal TNF-α expression is associated with a variety of pathologies, such as autoinflammatory diseases, tumors, and septic shock (55). Our studies proved that TNF-α can enhance the expression and secretion of ANGPTL8 and that the TNF-α antibody adalimumab can reverse the LPS-induced upregulation of ANGPTL8 and lipid deposition in HepG2 cells. *In vivo* studies revealed that although TNF-α receptor inhibitors improve liver lipid deposition and peroxidation, they do not improve the survival rate of mice with sepsis as much as knocking out *Angptl8* does. These results suggest that despite septic patients experiencing an inflammatory storm, anti-inflammatory therapy does not achieve the desired therapeutic effect. Furthermore, we also found that the expression of TNF-α in the livers of *Angptl8* KO septic mice was reduced, which may protect against liver tissue damage. Therefore, improved liver lipid metabolism alleviates liver damage and improves survival by reducing lipid deposition and inflammation, and ANGPTL8 monoclonal antibodies have attractive clinical application prospects.

Collectively, our results indicated that inflammation caused by infection leads to a disorder of liver lipid metabolism and that ANGPTL8 deficiency improves the survival rate of mice with sepsis by suppressing the inflammatory response and improving PGC1α/PPARα-mediated lipid metabolism. Inhibitors of ANGPTL8 may reduce organ damage and improve the prognosis of sepsis patients. However, our study mimics sepsis caused by bacterial infection using i.p. injection of LPS, a critical pathogen-associated molecular pattern involved in the pathogenesis of sepsis, and further validation of our conclusions through other sepsis models is needed. Since sepsis is a syndrome of multiple-organ dysfunction, further exploration of the role of ANGPTL8 in lung injury, kidney injury, and other organ injury has guiding significance for the development of ANGPTL8-targeted therapy.

## Data Availability

All relevant data supporting this study are contained within the article, in the supplemental data, or from the corresponding author upon request.

## Supplemental data

This article contains supplemental data.

## Conflict of interest

The authors declare that they have no conflicts of interest with the contents of this article.
